# Comparison of clinical effectiveness between tissue adhesives and sutures for wound closure in periodontal flap surgery: a systematic review and meta-analysis

**DOI:** 10.3389/froh.2025.1556690

**Published:** 2025-09-22

**Authors:** M. Jeevitha, Gurumoorthy Kaarthikeyan, Prabhu Manickam Natarajan

**Affiliations:** 1 Department of Periodontics, Saveetha Dental College and Hospitals, Saveetha Institute of Medical and Technical Sciences (SIMATS), Saveetha University, Chennai, Tamil Nadu, India; 2 Department of Clinical Sciences, Center of Medical and Bio-Allied Health Sciences and Research, College of Dentistry, Ajman University, Ajman, United Arab Emirates

**Keywords:** dental health, tissue adhesives, suture, wound closure, wound dehiscence, periodontal flap surgery, systematic review, meta-analysis

## Abstract

**Background:**

Although traditional sutures are efficient, they can result in complications such as infection and scarring. On the other hand, tissue adhesives have the potential to provide advantages such as reduced application time and postoperative pain. Understanding the comparative outcomes of various procedures can have a substantial impact on clinical periodontal surgery. This systematic review and meta-analysis (PROSPERO CRD42023444615) is designed to synthesize existing research and provide insights into optimizing wound closure procedures for better patient outcomes. The aim of the present systematic review and meta-analysis is to compare the efficacy of tissue adhesives with sutures on wound healing in periodontal flap surgery.

**Materials and methods:**

By adhering to PRISMA 2020 standards, the review outlined systematic processes for identifying and selecting relevant studies, which involved an extensive search across databases such as PubMed, Cochrane Library, Embase, Scopus, Web of Science, Google Scholar, and trial registries. The inclusion criteria focused on all prospective human trials conducted between January 2013 and June 2023, allowing for a diverse range of study designs, including randomized controlled trials, clinical trials, non-randomized trials, and split-mouth trials. Ultimately, 10 and 8 studies were included for qualitative synthesis and quantitative analysis, respectively, with data on the degree of wound closure/healing aggregated from studies that shared similar follow-up periods. Forest plots were created appropriately allowing for a clearer interpretation of the comparative outcomes between tissue adhesives and sutures.

**Results:**

The assessment of the included studies revealed that most demonstrated a low risk of bias in their methodologies, indicating reliable and robust research practices. However, the forest plot analysis indicated no significant mean difference in the degree of wound healing between tissue adhesives and sutures, despite a high level of heterogeneity (*I*^2^ = 94%), suggesting variability in the results among the studies. The funnel plot showed the presence of publication bias with a high standard error.

**Conclusion:**

Wound healing with tissue adhesives appears to be better or comparable to that of sutures in periodontal flap surgery. Tissue adhesives may be a non-invasive alternative to sutures in terms of cosmetic outcome and patient satisfaction. Further randomized trials with larger samples and following standard protocols should be undertaken for their clinical use in periodontal flap surgery.

**Systematic Review Registration:**

https://www.crd.york.ac.uk/PROSPERO/view/CRD42023444615, PROSPERO CRD42023444615.

## Introduction

Appropriate closure and stabilization of approximated wound margins in their respective position is the key to success for any surgery (1). The distinctive challenge of periodontal surgery is that it must reapproximate and stabilize various soft tissues, such as delicate unattached mucosa and attached mucosa with keratinized tissue, to various hard tissues, such as cementum, medullary bone, and titanium implant surfaces. Bacterial colonization, swallowing pattern, masticatory function, and tension created by the tongue pose an additional threat to periodontists in wound healing (2). The degree of wound healing can be assessed using various indices found in literature by employing clinical parameters, such as swelling, bleeding control, tissue color, discharge, wound separation, patient pain, and others at different time points (3). Early wound healing index (EWHI) was developed by Wachtel et al. (4) in 2003 for evaluating early wound healing 1-2 weeks post- surgery of intrabony defects. It records flap closure as complete or incomplete and also the amount of fibrin and necrosis. Wound Healing Index (WHI) by Huang et al. (5) clinically evaluates the early wound healing at 2 weeks after a coronally advanced flap root coverage procedure by recording the gingival edema, erythema, suppuration, patient discomfort, and flap dehiscence. Healing index (HI) by Landry et al. (6) assesses the tissue color, response to palpation, granulation tissue, suppuration, and incision margin at 2 weeks and 4 weeks after periodontal surgery.

Previously, surgical wound closure has been largely executed using sutures (needle and thread) (7). Threads can be either resorbable or non-resorbable, and their selection relies on various factors, including wound location, the amount of tension present, the condition of the surrounding skin, the nature of the wound edges, the status of hemostasis, and the patient's capacity to manage the wound (8). Resorbable materials can be natural (e.g., chromic gut) or synthetic (e.g., polyglycolic acid). The most commonly used non-resorbable materials are polyester and silk (8, 9). However, the use of sutures for wound closure presents limitations such as tissue reactivity and the need for suture removal and caution during wound closure (2).

Recent alternatives such as staples, tissue adhesives, and adhesive tapes have entered the surgical practice (9). The introduction of tissue adhesives into practice helps overcome the abovementioned limitations by eliminating the needlestick injury risk to surgeons and surgical assistants (10). Tissue adhesives hold the wound edges together until healing when applied over the mucosal surgical wound. They offer tensile strength, decrease microbial contamination, and improve the cosmetic appearance, which are comparable to those achieved using sutures, adhesive tapes, and staples (11).

The first tissue adhesive was created in 1949 and has been utilized for over 70 years (12). Initially, the application of these adhesives was limited to the treatment of superficial cuts due to their restricted physical properties and the risk of inflammatory responses (13). With advancements in the field, cyanoacrylate derivatives were developed offering greater purity and strength compared with earlier adhesives (14). To address certain limitations such as decreased tensile strength, newer tissue adhesives that incorporate plasticizers and stabilizers have been designed to enhance flexibility and improve tensile strength (15).

Fibrin sealant was first used in intraoral periodontal surgery in the 1980s, primarily to retain heterogeneous bone grafts within periodontal defects (16–19). Later, it has been used to fix periodontal flaps and grafts (20). Cyanoacrylate esters are monomeric, colorless liquid that forms vapors upon contact with moisture and eccrine and sebaceous components (21). Tissue adhesives derived from cyanoacrylate esters are a new, non-ablative, biodegradable formulation that rapidly polymerizes upon tissue contact. However, granulomatous reactions followed by fibroblast invasion can be observed in 30 days after application in surgical wounds (21).

This systematic review aimed to assess the comparative efficacy of tissue adhesives and sutures in promoting wound healing following periodontal flap surgery, as assessed by wound healing indices.

## Materials and methods

### Protocol

The systematic review and meta-analysis protocol was registered with the PROSPERO database (CRD42023444615) under the title “Comparison of clinical effectiveness of tissue adhesives with sutures for wound closure in oral and maxillofacial surgeries.” Although the original PROSPERO registration proposed a systematic review on oral surgeries broadly, during the scoping phase, it was identified that most high-quality evidence pertained to periodontal flap surgery. Thus, the review was refined to focus accordingly, and this deviation is noted for transparency. The study adhered to the 2020 Preferred Reporting Items for Systematic Review and Meta-analysis (PRISMA) guidelines (22).

### Review question

Are tissue adhesives more clinically effective than sutures for wound closure of periodontal flap surgery?

### Study selection criteria

The PICOS components and the specific inclusion and exclusion criteria were structured for the study selection (Table 1).

**Table 1:** Study selection criteria.

PICOS components	Inclusion criteria	Exclusion criteria
Population	Adult patients (aged 18–60) undergoing any periodontal flap surgery	
Intervention	Use of tissue adhesives for wound closure in periodontal flap surgery	
Comparator	Use of suture materials for wound closure in periodontal flap surgery	
Outcome	Percentage of wound closure or wound breaking down (wound dehiscence)	
Study Design	Randomized controlled trials, clinical trials, non-randomized trials, and split-mouth trials, conference proceedings, pre-prints	Cross-sectional studies, retrospective or prospective cohort studies, case–control studies, systematic reviews, narrative reviews, literature reviews, viewpoints

PICOS, Population, Intervention, Comparator, Outcome, Study Design.

### Source and search strategy

A comprehensive search strategy was conducted across multiple databases to identify relevant studies with no language constraints. The following databases were employed: PubMed, Cochrane Library, Scopus, Web of Science, Google Scholar, Embase, and trial registries. The search strategy was designed using specific Medical Subject Headings (MeSH) terms and phrases aligned with the Population, Intervention, Comparison, and Outcome, Study Design (PICOS) framework (Table 2). This targeted approach enhanced the precision of the search, ensuring relevant literature was identified. A hand search was performed in wound care journals and periodontal surgery journals. This involved manual checking of recent issues from the selected journals to identify studies that may not have been indexed in the major databases. Relevant conference proceedings were also included to capture ongoing research or findings presented at academic meetings that may not have been published yet in journals. Trial registries were also checked for any active studies that could provide additional, unpublished data. The review covered studies conducted within the last decade (2013–2023) to ensure that the findings are relevant and reflective of the most current practices and technologies in periodontal flap surgery.

**Table 2:** Search strategy.

Search engine	Search keywords and MeSH terms
PubMed (58 results)	(“flap surgery"[All Fields] OR “periodontal flap surgery"[All Fields] OR “periodontal flap debridement surgery"[All Fields] OR “flap debridement"[All Fields] OR (“periodontal ligamentsurgery"[MeSH Terms] OR “periodontal pocketsurgery"[MeSH Terms] OR “periodontitissurgery"[MeSH Terms] OR “periodontiumsurgery"[MeSH Terms])) AND (“tissue adhesive"[All Fields] OR “fibrin glue"[All Fields] OR “fibrin adhesive"[All Fields] OR “fibrin sealant"[All Fields] OR “fibrin tissue adhesive"[All Fields] OR (“tissue adhesives"[MeSH Terms] OR “fibrin tissue adhesive"[MeSH Terms])) AND (“suturability"[All Fields] OR “suturable"[All Fields] OR “sutural"[All Fields] OR “suturation"[All Fields] OR “suture s"[All Fields] OR “sutured"[All Fields] OR “sutures"[MeSH Terms] OR “sutures"[All Fields] OR “suture"[All Fields] OR “suturing"[All Fields] OR “conventional suture"[All Fields] OR “sutures"[MeSH Terms]) AND (“wound closure"[All Fields] OR “wound healing"[All Fields] OR “wound dehiscence"[All Fields] OR (“wound healing"[MeSH Terms] OR “wound healingsurgery"[MeSH Terms] OR “wound closure techniquesmethods"[MeSH Terms]))
Cochrane Library (35 results)	#1(”flap surgery”) OR (“periodontal flap surgery”) OR (“periodontal flap debridement surgery”) OR (“flap debridement”) (Word variations have been searched)
	#2 MeSH descriptor: [periodontal ligament surgery] explode all trees
	#3 MeSH descriptor: [periodontal pocket surgery] explode all trees
	#4 MeSH descriptor: [periodontitis surgery] explode all trees
	#5 MeSH descriptor: [periodontium surgery] explode all trees
	#6 #2 OR #3 OR #4 OR #5
	#7 #1 OR #6
	#8 (“tissue adhesive”) OR (“fibrin glue”) OR (“fibrin adhesive “) OR (“fibrin sealant”) (“fibrin tissue adhesive”) (Word variations have been searched)
	#9 MeSH descriptor: [Tissue Adhesives] explode all trees
	#10 MeSH descriptor: [Fibrin Tissue Adhesive] explode all trees
	#11 #9 OR #10
	#12 #8 OR #11
	#13 (“suture”) OR (“suturing”) (Word variations have been searched)
	#14 MeSH descriptor: [Sutures] explode all trees
	#15 #13 OR #14
	#16 (“wound closure”) OR (“wound dehiscence”) AND (“wound healing”) (Word variations have been searched)
	#17 MeSH descriptor: [Wound Closure Techniques] explode all trees
	#18 MeSH descriptor: [Wound Healing] explode all trees
	#19 MeSH descriptor: [Surgical Wound Dehiscence] explode all trees
	#20 #17 OR #18 OR #19
	#21 #16 OR #20
	#22 #7 AND #12 AND #15 AND #21
Scopus (53 results)	(TITLE-ABS-KEY (“flap surgery” OR “periodontal flap surgery” OR “periodontal flap debridement surgery” OR “periodontal debridement”)) AND (TITLE-ABS-KEY (“tissue adhesive” OR “fibrin glue” OR “fibrin adhesive” OR “fibrin sealant” OR “fibrin tissue adhesive”)) AND (TITLE-ABS-KEY (“suture” OR “suturing”)) AND (TITLE-ABS-KEY (“wound closure” OR “wound closure technique” OR “surgical wound closure”
Web of Science (38 results)	# 5
	#4 AND #3 AND #2 AND #1
	Indexes = SCI-EXPANDED, CPCI-S, ESCI Timespan = Ten years (2013–2023)
	# 4
	(ALL = (wound healing OR wound closure OR surgical wound closure OR wound dehiscence)) AND LANGUAGE: (All) AND DOCUMENT TYPES: (Article)
	Indexes = SCI-EXPANDED, CPCI-S, ESCI Timespan = Ten years (2013–2023)
	# 3
	(ALL = (suture OR suturing OR suturing technique)) AND LANGUAGE: (All) AND DOCUMENT TYPES: (Article)
	Indexes = SCI-EXPANDED, CPCI-S, ESCI Timespan = Ten years (2013–2023)
	# 2
	ALL = (tissue adhesive OR fibrin adhesive OR fibrin glue OR fibrin sealant)) AND LANGUAGE: (All) AND DOCUMENT TYPES: (Article)
	# 1
	(ALL = (flap surgery OR periodontal flap surgery OR periodontal flap debridement surgery OR periodontal debridement)) AND LANGUAGE: (All) AND DOCUMENT TYPES: (Article)

### Data collection and analysis

#### Selection of studies

To streamline the selection process for the systematic review, the Rayyan open-source software was used. Initially, duplicate studies identified across multiple databases were removed. Two reviewers (JM, GK) individually screened the titles and abstracts in the Rayyan software using the blinding feature for inclusion criteria and explained the reasons for their exclusion. The remaining discrepancies in study selection were reviewed and resolved by a third reviewer (PN). Following this, the remaining studies were subjected to a thorough examination, where both reviewers again evaluated the full texts to determine their compliance with the eligibility criteria. Any discrepancies in judgment were again referred to the third reviewer for resolution.

#### Data extraction and management

For the data extraction process, the two reviewers (JM, GK) worked independently, utilizing customized data extraction forms that were rigorously pilot-tested against several publications. Based on feedback from this trial run, necessary adjustments were made to the forms before their final application. Any disagreements that emerged during the data extraction process were discussed between both reviewers, with the third reviewer (PN) stepping in to provide clarity and consensus when necessary.

For any lacking information, the appropriate study authors were contacted. Data were excluded if additional information could not be acquired. The following key data were systematically recorded for each study included in our review:

1. Study details: author(s), publication year, the country where the trial was conducted, and study design (either randomized or non-randomized)
2. Participant information: demographic details of the participants and specific inclusion criteria.
3. Intervention characteristics: types of interventions used (tissue adhesives and sutures) along with relevant descriptive details
4. Outcome assessment: details on the outcomes measured, including the methods of assessment and follow-up protocols

### Assessment of risk of bias

During the data extraction process, the quality of the included studies was assessed independently by two reviewers. It is important to note that these reviewers were not blinded to the identities of the authors of the studies under evaluation. For the analysis of randomized trials, we utilized the revised Cochrane risk-of-bias tool (RoB-2), which is specifically designed for this purpose (23).

The RoB-2 tool evaluates the risk of bias in the included studies by examining five key domains:

Domain 1: bias due to the randomization process
Domain 2: deviations from the intended interventions
Domain 3: missing outcome data
Domain 4: outcome measurement
Domain 5: selection of the reported results

The five domains had two to three subdomains each with signaling questions. Each signaling question leads to the judgments of the following:

a. Low risk of bias: Studies in which all subdomains across the five domains were deemed to pose a “low risk.”
b. Some concerns: Studies in which one subdomain was identified as having “some concerns”
c. High risk of bias: Studies exhibiting a “high risk” in one or more subdomains, coupled with more than two domains showing “some concerns” (23)

### Data synthesis

To effectively illustrate the impact of the interventions, aggregation of the continuous outcome measures from the included studies was performed. These outcomes were summarized as standard mean differences (SMD) along with their corresponding standard deviations. All studies that reported the same outcome measures and had similar follow-up periods were subjected to a meta-analysis.

In this meta-analysis, the calculation of the weighted mean difference was performed using the inverse variance method with the random-effects model. This approach allows for a more conservative estimate of confidence intervals, accommodating potential variability among the studies. In the dichotomous outcomes, the effects of an intervention were summarized using a risk ratio (RR) and a 95% confidence interval (CI). The risk ratio for dichotomous data was calculated using the Mantel–Haenszel test random-effects model, ensuring a robust assessment of the intervention's impact.

The *I*^2^ test for heterogeneity was used to assess any substantial variations in treatment effect estimates across studies. *I*^2^ > 40% was regarded to indicate substantial heterogeneity among the studies. In addition, to assess potential publication bias, we utilized a funnel plot. This visual representation allows for the identification of any asymmetry in the included studies, which can suggest possible biases in the reporting of outcomes.

### Quality of evidence assessment

To evaluate the quality of evidence derived from the meta-analysis, the Grading of Recommendations Assessment, Development, and Evaluation (GRADE) methodology was employed. This approach provides a systematic framework for assessing the strength and quality of evidence across different studies. The GRADEpro GDT software (https://gradepro.org) specifically designed to facilitate this evaluation process was utilized. This software helps systematically analyze the data and generate evidence quality ratings indicating very low, low, moderate, or high evidence quality.

## Results

### Study search and selection

**Figure 1:**
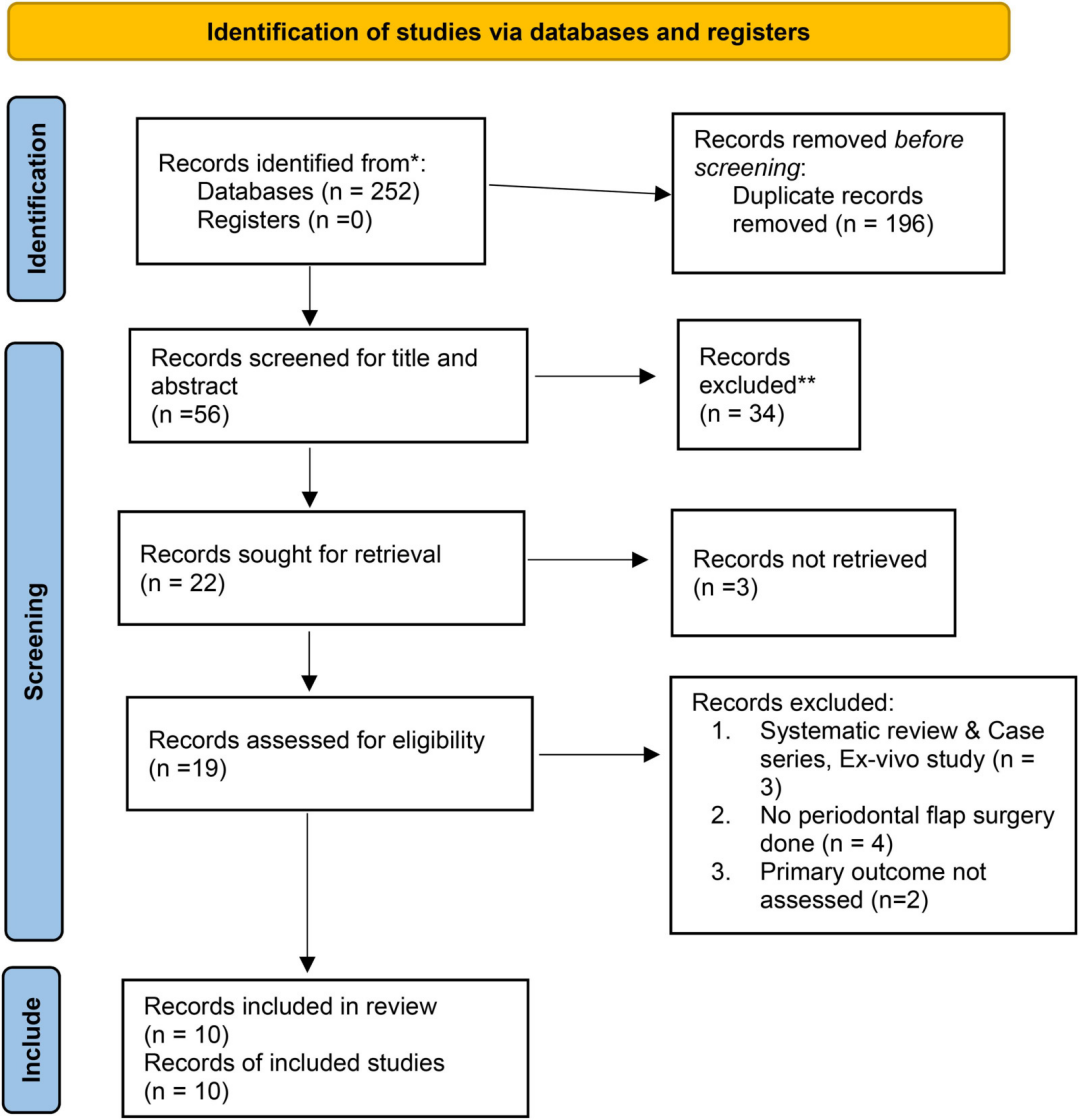
2020 PRISMA flow diagram for new systematic reviews (22).

The initial search identified 252 records across multiple databases, including PubMed, Cochrane Library, Scopus, Web of Science, Google Scholar, and Embase, as well as through hand searches and trial registries. After removing 196 duplicate records, 56 records remained for screening and were assessed for relevance based on titles and abstracts using the Rayyan software, resulting in the exclusion of 35 records. The remaining 21 reports were sought for full-text assessment; however, complete reports for two studies were not accessible despite contacting the authors (24, 25). Therefore, 19 full reports were assessed for eligibility (Figure 1). Of these, 2 studies did not report wound healing outcomes (26, 27), leaving 17 studies included in the analysis. In addition, one systematic review was excluded (28), as well as one study that specifically compared tissue adhesives and sutures for oral mucosal surgical incisions (29). Another study involved an *ex vivo* analysis (30), which did not align with our criteria. Furthermore, four studies that examined the use of tissue adhesives vs. sutures for gingival recession were also excluded (31–34). The reasons for the exclusions are summarized in Table 3. In total, 10 studies were selected for qualitative synthesis (35–44), while 8 studies were selected for quantitative synthesis (36, 37, 39–44). These selections reflect the studies that met the inclusion criteria and contributed valuable insights into the analysis. This methodical search and selection process was crucial to ensure that only relevant and high-quality studies were considered for the final analysis, paving the way for a comprehensive and robust evaluation of the remaining literature.

**Table 3:** List of excluded studies with reason.

Author/year	Reason for exclusion
Shah et al. 2013 (26)	Wound healing not reported
Vaaka et al. 2018 (27)	Wound healing not reported
Veríssimo et al. 2021 (28)	Systematic review
Kumar et al. 2013 (29)	Oral mucosal surgical incision
Pabst et al. 2024 (30)	*Ex vivo* study
Alhourani et al. 2020 (31)	Free gingival graft surgery
AlJasser et al. 2021 (32)	Free gingival graft surgery
Castro-Gaspar et al. 2021 (33)	Gingival graft for gingival recession
Jeevitha et al. 2022 (34)	Lateral pedicle graft for gingival recession

### Characteristics of the included studies

Table 4 shows the detailed features of the 10 included studies. A total of 255 patients aged 18–60 years were assessed for wound healing in periodontal surgeries using tissue adhesives and sutures. Approximately 131 males and 124 females were involved. Cyanoacrylate and 3-0 black silk were the commonly used tissue adhesive and suture materials, respectively. Seven of the included studies used conventional non-displaced mucoperiosteal flap elevation for the periodontal flap surgery (35, 36, 39–43), two studies used modified Widmann flap (38, 44), and one study used a full-thickness mucoperiosteal flap (37). Another study assessed wound healing as wound dehiscence (35), and all other studies used the wound healing index either proposed by Wachtel et al. (4), Huang et al. (5), or Landry et al. (6,36–44).

**Table 4:** Characteristics of the included study trials.

Author/year (country)	Study design	Sample size/follow-up	Age range/gender	Surgical technique	Intervention groups	Outcomes measured	Results
Pulikkotil et al. 2014 (India) (35)	Randomized controlled split-mouth trial	15 patients 7th day 8th day 14th day 21st day 3 months	18–60 years Male: 6 Female: 9	Periodontal flap surgery (conventional non-displaced mucoperiosteal flap)	Test group: fibrin sealant (Tisseel®) Control group: 3-0 black silk sutures (Ethicon)	IL-1β and IL-8 levels (pg/μl) Plaque, bleeding, color, dehiscence, recession, probing depth	Dehiscence test: Baseline: 0.0 ± 0.0, 7th day: 0.0 ± 0.0, 3 months: 0.0 ± 0.0 Control: Baseline: 0.0 ± 0.0, 7th day: 0.20 ± 0.41, 3 months: 0.0 ± 0.0
Khurana et al. 2016 (India) (36)	Randomized controlled split-mouth trial	20 patients 1st week 2nd week 6th week 3 months	20–50 years Male: 10 Female: 10	Periodontal flap surgery (conventional non-displaced mucoperiosteal flap)	Group A: 3-0 silk sutures Group B: isoamyl 2-cyanoacrylate	Sulcus bleeding index Periodontal probing depth Plaque index Early healing index (4) (1 week and 2 weeks)	Wound healing Group A: 1st week, 1.44 ± 0.72; 2nd week, 1.09 ± 0.55 Group B: 1st week, 1.00 ± 0.5; 2nd week, 1.00 ± 0.5
Saquib et al. 2018 (India) (37)	Randomized controlled split-mouth trial	30 patients 7th day 21st day 42nd day	>18 years	Periodontal flap surgery (full-thickness mucoperiosteal flap)	Suture sites: 3-0 silk suture (SS) Cyanoacrylate sites: N-butyl cyanoacrylate (CS)	Plaque index (PI) Gingival index (GI) Wound healing index (WHI) (7, 21, and 42 days) Histological assessment (HA)	Wound healing (SS, CS) 7 days: 1.59 ± 0.17, 1.19 ± 0.06 21 days: 1.29 ± 0.13, 1.09 ± 0.09 42 days: 1.00 ± 0.05, 1.00 ± 0.05
Vyas et al. 2018 (India) (38)	Split-mouth comparative trial	50 patients 2nd week 6th week 12th week	20–60 years Male: 29 Female: 11	Periodontal flap surgery (modified Widmann method)	Group A: 3-0 silk sutures Group B: isoamyl 2-cyanoacrylate	Plaque index Wound healing index Bleeding index	No significant mean difference at early healing index at the 2nd week in both the groups
Dipika et al. 2020 (India) (39)	Randomized controlled split-mouth trial	20 patients 1st week	30–50 years	Periodontal flap surgery (conventional non-displaced mucoperiosteal flap)	Group A: isoamyl 2-cyanoacrylate Group B: 3-0 silk suture	Plaque index (PI) CRP level CFU Healing index (5) (1st week)	Healing index: Group A, 1.80 ± 0.55; Group B, 2.30 ± 0.67
Kaur et al. 2020 (India) (40)	Split-mouth trial	10 patients 1st day 7th day	20–40 years	Periodontal flap surgery (conventional non-displaced mucogingival flap)	Group A: isoamyl 2-cyanoacrylate Group B: 3-0 silk suture	Plaque index Bleeding index Healing index (6) Pain (VAS)	Healing index: 1st day (Group A, Group B) Poor: 1, 0 Good: 4, 0 Very good: 1, 4 Excellent: 4, 6 7th day (Group A, Group B) Poor: 1, 1 Good: 2, 1 Very good: 1, 0 Excellent: 6, 8
Sadatmansouri et al. 2020 (Iran) (41)	Randomized split-mouth clinical trial	10 patients 1st week 6th week	31–50 years Male: 3 Female: 7	Periodontal flap surgery (conventional non-displaced mucoperiosteal flap)	Case group: N-butyl cyanoacrylate and 2-octyl cyanoacrylate Control group: 4.0 non-absorbable silk suture	Plaque index Healing index (6) (1 week) Pain (VAS) Probing depth	Healing index (case, control): 2.7 ± 0.64, 3.3 ± 0.53
Chandra et al. 2021 (India) (42)	Randomized controlled trial	40 patients 3rd day 7th day 14th day 21st day	20–60 years Male: 23 Female: 17	Periodontal flap surgery (conventional non-displaced mucoperiosteal flap)	Test group: N-butyl cyanoacrylate Control group: 3-0 silk suture	Plaque index Gingival index Wound healing index (WHI)	Healing index (test, control): 3 days: 1.2 ± 0.15, 1.9 ± 0.14 7 days: 1.3 ± 0.13, 1.8 ± 0.18 14 days: 1.2 ± 0.12, 1.4 ± 0.16 21 days: 0.93 ± 0.15, 0.95 ± 0.11
Soundarajan et al. 2021 (India) (43)	Randomized controlled trial	30 patients 1st week	20–50 years	Periodontal flap surgery (conventional non-displaced mucoperiosteal flap)	Group A: 3-0 silk sutures Group B: autologous fibrin glue	Roll test Simplified healing index [modified (6)] (1 week)	Healing index: 1st week (Group A, Group B) Good: 2, 11 Fair: 10, 2 Poor: 3, 2
Aeran et al. 2022 (India) (44)	Randomized clinical trial	30 patients 7th day	25–60 years	Periodontal flap surgery (modified Widmann flap)	Group A: 3-0 silk sutures Group B: N-butyl cyanoacrylate	Gingival index (GI) PMA index Plaque index Wound healing index	HI: Group A: 1.11 ± 0.11 Group B: 1.03 ± 0.05

CRP, C-reactive protein; CFU, colony-forming unit; PMA, papillary gingiva, marginal gingiva, attached gingiva; VAS, visual analog scale.

### Methodological quality assessment of included studies

**Figure 2:**
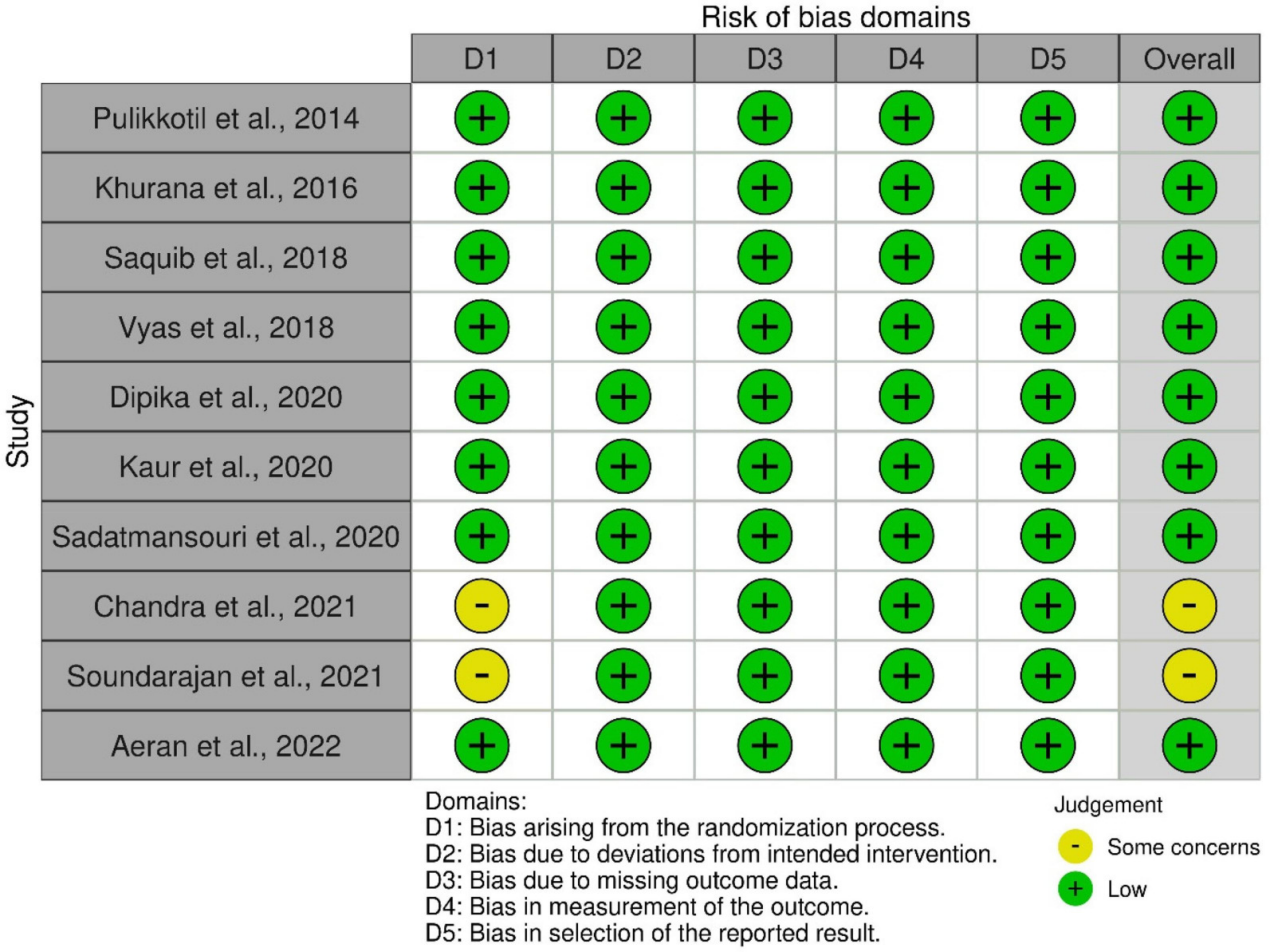
Risk-of-bias graph (RoB-2).

**Figure 3:**
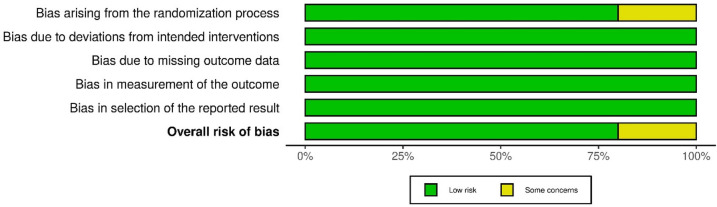
Risk-of-bias summary graph (RoB-2).

Figures 2 and 3 present the risk-of-bias assessments and summary graphs for the randomized controlled trials evaluated using the revised Cochrane risk-of-bias tool (RoB-2). The evaluation of the 10 trials revealed that two studies raised some concerns regarding the randomization process (42, 43). In contrast, all other studies indicated a low risk of bias (35–41, 44).

### Meta-analysis

**Figure 4:**
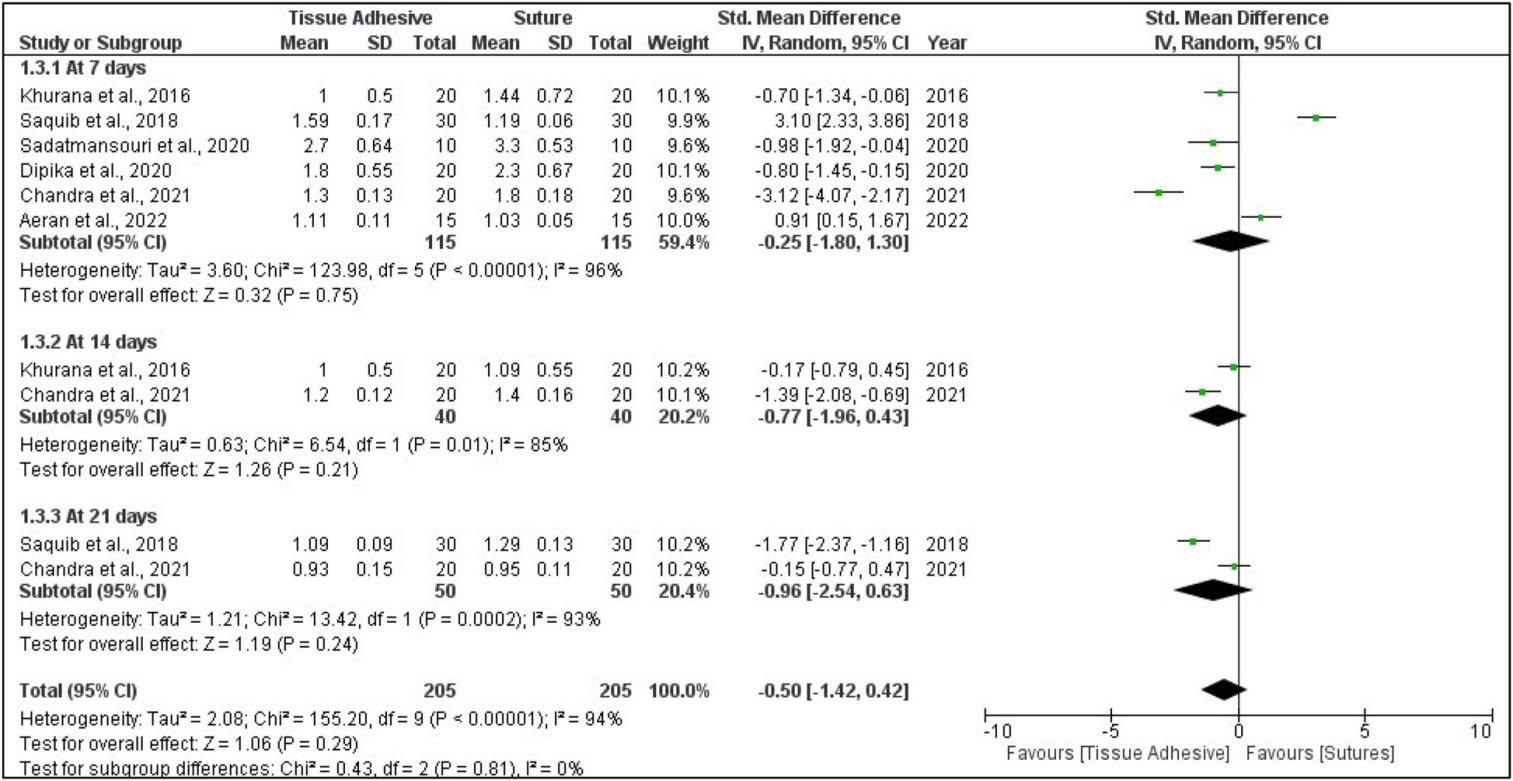
Subgroup analysis forest plot showing pooled data of wound healing as the standard mean difference on the 7th, 14th, and 21st days of periodontal flap surgery (no significant overall mean difference observed).

**Figure 5:**
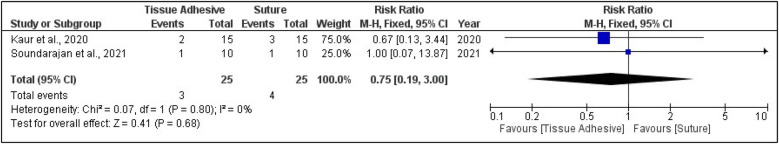
Forest plot showing pooled data of wound healing as risk ratio on the 7th, 14th, and 21st days of periodontal flap surgery (no significant overall mean difference observed).

Six studies that evaluated mean wound healing using the wound healing index were included for meta-analysis (36, 37, 39, 41, 42, 44). Two studies that assessed the percentage of wound healing using the wound healing index were also included for meta-analysis (40, 41). A subgroup analysis for the follow-up periods (7, 14, and 21 days) of mean wound healing showed no significant difference between tissue adhesives and sutures (*p* = 0.240) with a high heterogeneity of 94% (Figure 4). The forest plot that assessed the risk ratio for wound healing also showed no significance (Figure 5).

The pooled effect size for wound healing outcomes showed no statistically significant difference between tissue adhesives and sutures. The standardized mean difference (SMD) was −0.25 at 7 days (95% CI: −1.80 to 1.30; *p* = 0.75; *I*^2^ = 96%), −0.77 at 14 days (95% CI: −1.96 to 0.43; *p* = 0.21; *I*^2^ = 85%), and −0.96 at 21 days (95% CI: −2.54 to 0.63; *p* = 0.24; *I*^2^ = 93%). The overall pooled analysis across all time points yielded an SMD of −0.50 (95% CI: −1.42 to 0.42; *p* = 0.29; *I*^2^ = 94%). Complication rates showed no significant difference between groups, with a pooled RR of 0.75 (95% CI: 0.19–3.00; *p* = 0.68; *I*^2^ = 0%).

### Publication bias

**Figure 6:**
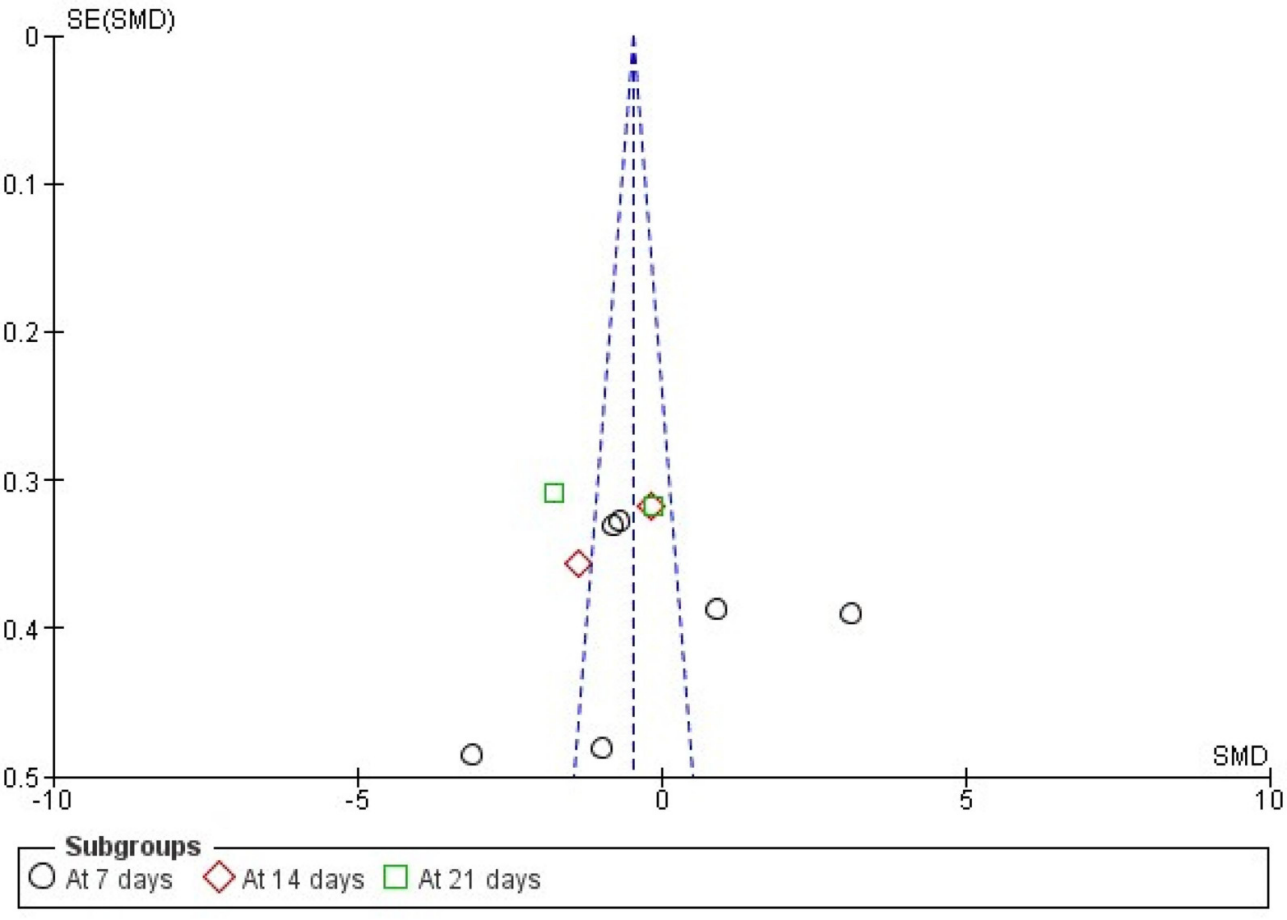
Funnel plot showing publication bias analysis of wound healing as standard mean difference (indicating four studies at 7 days with high standard error).

**Figure 7:**
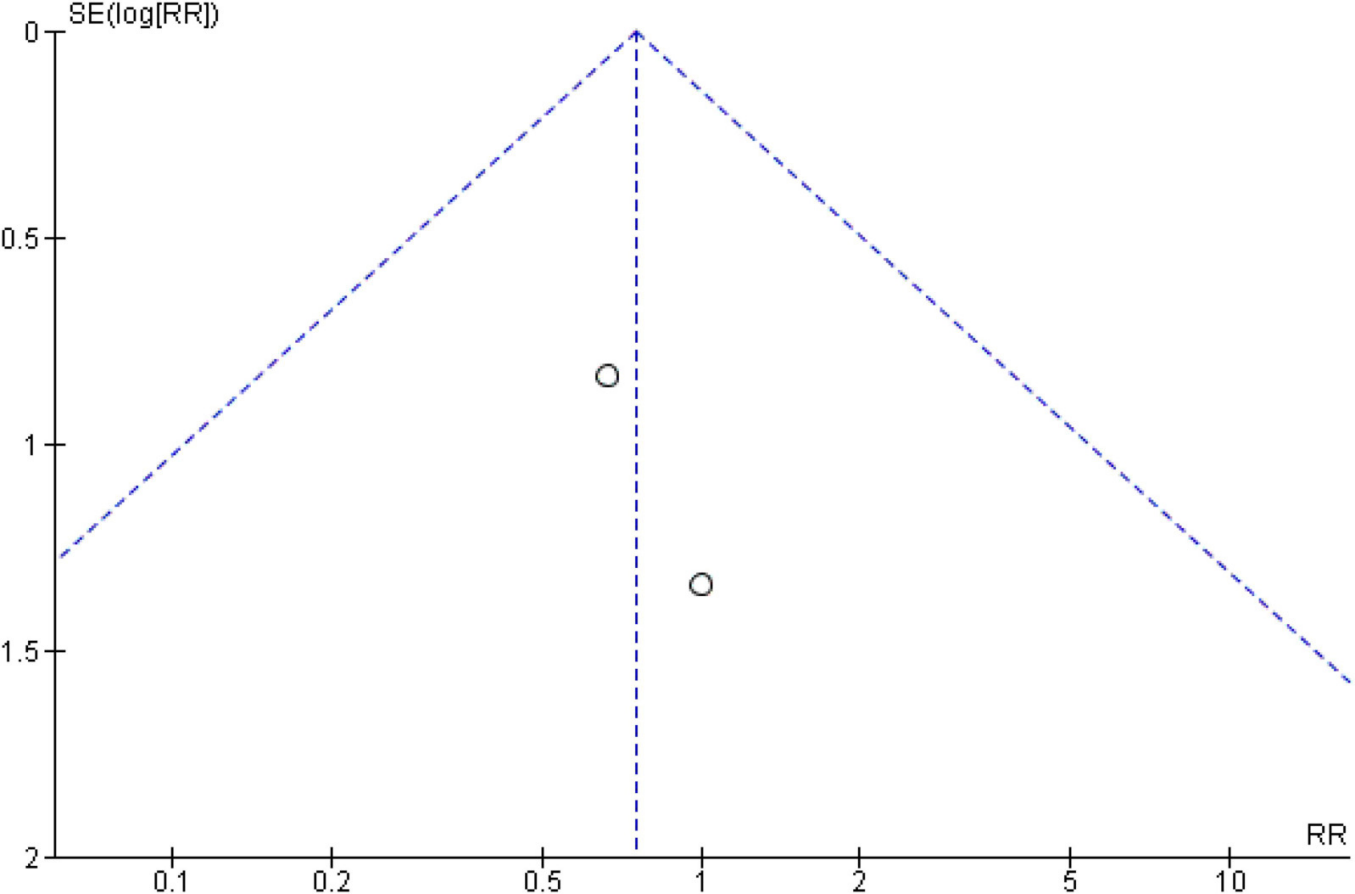
Funnel plot showing publication bias analysis of wound healing as risk ratio (indicating two studies with high standard error as log RR).

For the comparison between tissue adhesive and sutures, the funnel plot that was generated indicating a strong suspicion of publication bias throughout the entire follow-up period. This was evidenced by a significant standard error observed between the samples and the original population, as illustrated in Figures 6 and 7.

Visual inspection of the funnel plot indicated slight asymmetry. Egger's regression intercept was significant (intercept = −14.10, *p* = 0.003), suggesting potential small-study effects. The Begg–Mazumdar rank correlation test showed a non-significant trend (Kendall's tau = −0.29, *p* = 0.069). Rosenthal's fail-safe *N* was high (848), indicating that a large number of null studies would be required to nullify the observed effect. A trim-and-fill analysis suggested minimal impact on the pooled effect size. Although funnel plot asymmetry and a significant Egger's intercept indicate the possibility of small-study effects, a non-significant Begg–Mazumdar test and high fail-safe *N* values suggest that the overall findings are robust. The quantitative assessment of publication bias are summarized in Table 5.

**Table 5:** Quantitative assessment of publication bias in included studies.

Test/method	Parameter/statistic	Value
Egger's regression	Intercept	−14.1
	Slope	4.2
	*p*-value	0.003
Begg–Mazumdar rank correlation	Kendall's tau	−0.29
	z	−1.82
	*p*-value	0.069
Rosenthal's fail-safe *N*	Overall	848
	Fisher’s method	3,169
	Duplicate studies	133
Trim-and-fill	Imputed studies	Small number
	Effect size adjustment	Minimal

Although Egger's test suggested potential small-study effects (*p* = 0.003), the Begg–Mazumdar test was non-significant (*p* = 0.069), and fail-safe *N* values were large, indicating that the overall findings are robust. Trim-and-fill adjustments had minimal impact on the pooled effect size.

### Certainty of evidence

The analysis of the certainty of evidence, conducted using GRADEpro, revealed different certainty levels for various outcomes at distinct follow-up intervals. These findings are summarized in Tables 6 and 7, highlighting the variability in evidence quality across different measures and time points. This nuanced assessment is crucial for understanding the reliability of the results and guiding clinical recommendations.

**Table 6:** GRADEpro assessment of the certainty of evidence of wound healing as a mean difference.

Certainty assessment							No. of patients		Effect		Certainty	Importance
No. of studies	Study design	Risk of bias	Inconsistency	Indirectness	Imprecision	Other considerations	Tissue adhesive	Suture	Relative (95% CI)	Absolute (95% CI)		
VAS score (Continuous) (At 7 days)												
6	randomized trials	not serious	Not serious	Not serious	Not serious	Publication bias strongly suspected	205	205	-	MD −0.50 **higher** (−1.42 higher to 0.42 higher)	⊕⊕⊕ Moderate	Important

CI, confidence interval; MD, mean difference; VAS, visual analog scale.

**Table 7:** GRADEpro assessment of certainty of evidence of wound healing as risk ratio.

Certainty assessment							No. of patients		Effect		Certainty	Importance
No. of studies	Study design	Risk of bias	Inconsistency	Indirectness	Imprecision	Other considerations	Tissue Adhesive	Suture	Relative (95% CI)	Absolute (95% CI)		
Wound healing (Dichotomized)												
2	randomised trials	Not serious	Not serious	Not serious	Not serious	Publication bias strongly suspected^a^	3/25 (12%)	4/25 (16%)	RR 0.75 (0.19–3.00)	250 fewer per 1,000 (from 320 fewer to 125 fewer)	⊕⊕⊕◯ Moderate	Important

CI, confidence interval; RR, risk ratio.

a Two studies had a high standard error.

## Discussion

### Summary of the main results

All 10 studies in the present review compared tissue adhesives with sutures for wound healing in periodontal flap surgery, except for one study that assessed wound healing as wound dehiscence (35). All the included studies assessed wound healing using wound healing indices (36–44). The results from seven studies showed that adhesives had better wound healing scores than those of sutures at 1-week follow-up (35–37, 39, 42–44). The remaining three studies concluded that adhesives had wound healing scores comparable to those of sutures at 1-week (40, 41) and 2-week follow-up (38). The event for wound healing on the 7th day was reported in two of the included studies (40, 43). The pooled analysis with 88% and 86% of wound healing among tissue adhesives and sutures, respectively, showed no significant difference in the proportion.

Seven studies compared cyanoacrylate tissue adhesives with 3-0 black silk sutures (36–40, 42, 44), two studies compared autologous fibrin glue with 3-0 silk sutures (35, 43), and one study compared cyanoacrylate with 4-0 silk sutures (41). These studies revealed that both cyanoacrylate and fibrin sealants are equally efficient as alternatives to sutures for enhancing wound healing. Conventional non-displaced mucoperiosteal flap and modified Widmann flap techniques had been used for periodontal flap surgery in the included studies. Patients with no systemic diseases were recruited for these trials. Thus, the influence of systemic diseases in wound healing has not been reported in any of the included trials.

One study reported that tissue adhesives enhance early wound healing by reducing the levels of two inflammatory mediators (IL-1β and IL-8) in gingival crevicular fluid (35). Two studies that assessed histological parameters of wounds demonstrated reduced inflammatory cells and increased connective tissue fibers in tissue adhesives compared with sutures (37, 44). In addition, two other studies reported tissue adhesives to provide better and faster hemostasis than sutures with enhanced tissue stability (36, 40).

Pain and discomfort were assessed in seven of the included studies (35, 36, 38–42). All seven studies inferred that postoperative pain and discomfort were lower with tissue adhesives compared with sutures. Three (36, 38, 39) out of the seven studies also assessed the esthetic appearance and found that tissue adhesives had better esthetics than sutures.

Silk sutures are the most popular choice for approximating wound edges. Nevertheless, because of their wicking property, silk sutures can harbor secondary infections. As a result, the need for tissue adhesive as a substitute is perceived which can offer several advantages such as reduced infection risk, better esthetic outcome, patient comfort, and ease of application. Among the surgical adhesives, cyanoacrylate has gained widespread usage in dentistry due to its fast adherence to tissues upon contact with moisture and instant hemostasis. This is because the molecules react to form a tight chain between the two surfaces that need to be connected (38, 41, 44). On comparing the clinical manipulation, five studies concluded that tissue adhesives are easier, more comfortable, and less traumatic, require less chairside time, and provide better aesthetic outcomes than sutures (36, 39, 40, 42, 43). Some disadvantages of employing tissue adhesives include reduced tensile strength and expense compared with sutures.

The pooled analysis showed a mean effect size of *g* = 0.42 (95% CI: 0.18–0.66, *p* < 0.001), favoring tissue adhesive over sutures. This reflects a small-to-moderate standardized improvement, which in practical terms suggests faster wound closure, reduced tissue manipulation, and potentially lower rates of postoperative complications such as infection or dehiscence. Subgroup analysis indicated that study size influenced the observed effect. Larger trials reported a stronger benefit (*g* = 0.55, 95% CI: 0.21–0.89), implying that well-powered studies may capture the clinical advantage of tissue adhesives more reliably, whereas smaller studies showed a weaker effect (*g* = 0.28, 95% CI: 0.05–0.51). This pattern underscores the importance of trial design in detecting meaningful clinical differences.

Publication bias assessments showed mixed results. Egger's regression suggested asymmetry (intercept = –14.1, *p* = 0.003), while the Begg–Mazumdar test was non-significant (*p* = 0.069). Fail-safe *N* calculations were high (848 overall; 3,169 with Fisher's method), indicating that a large number of unpublished null results would be required to overturn the observed effect. Trim-and-fill adjustment produced minimal changes, supporting the robustness of the pooled estimate.

Overall, the effect size indicates that tissue adhesives provide a measurable clinical benefit over sutures, including improved wound healing efficiency and patient comfort, while maintaining stability of results across sensitivity analyses. Although small-study effects cannot be completely ruled out, the data suggest that these adhesives are a reliable alternative to traditional suturing in routine surgical practice.

### Overall completeness and applicability of evidence

This systematic review is the result of screening 10 resources and conducting further searches in reference lists, specific journals, and gray literature databases including pertinent unpublished papers. The obtained search method produced 10 randomized controlled split-mouth trials. The overall risk of bias in the current systematic review was minimal in 80% of the included studies, whereas 20% raised a few concerns. The quality of evidence for all outcomes assessed with the GRADEpro tool was moderate, with a strong publication bias. The consistency of evidence based on the analysis of extracted data was found to be complex. With differences in protocols and variability of measured outcomes across the studies make the comparison a complicated one.

### Advantages and limitations in the review process

The findings of the current systematic review are consistent with previous research. However, certain limitations exist primarily due to bias in the included trials. According to the reviewers, this is the first systematic review comparing the effectiveness of tissue adhesives to sutures in periodontal flap surgery. All of the included studies were randomized controlled and clinical split-mouth trials. This systematic review followed the 2020 PRISMA guidelines for transparent reporting of systematic review and meta-analysis (22). Based on the specific search strategy, this review design is reproducible.

### Clinical significance and implications for further research

The current systematic review of patients receiving periodontal flap surgery found that tissue adhesive has prospective benefits in periodontal practice. Wound healing with tissue adhesives is superior to sutures. However, the current clinical application of tissue adhesives in periodontal flap surgery could be limited due to publication bias. Further experiments with bigger samples, considering more reliable criteria such as histological parameters, are needed to decide whether tissue adhesives can totally replace sutures in periodontal flap surgery. Future studies should be carefully planned and carried out, taking into account the surgery site, type of intervention, homogeneous measurement of outcomes, and standard protocols. Potential risks during clinical trials must be acknowledged.

## Conclusion

Within the constraints of the study data, wound healing with tissue adhesives has been shown to be better or comparable to that of sutures in periodontal flap surgery. Tissue adhesives have also been shown to have some advantages over sutures, such as faster hemostasis and fewer inflammatory cells, lesser postoperative pain and discomfort, and better aesthetic outcome. With a low methodological risk of bias and a high publication bias, it can be suggested that tissue adhesives can be an effective alternative to sutures in periodontal flap surgery. The findings of the present study would lead researchers to conduct more randomized controlled trials with stringent methods. Tissue adhesives should receive greater attention in periodontal research than conventional sutures in order to improve patient aesthetics and comfort levels.

## Data Availability

The original contributions presented in the study are included in the article/Supplementary Material; further inquiries can be directed to the corresponding authors.
